# Influence of Biofillers on the Properties of Regrind Crystalline Poly(ethylene terephthalate) (CPET)

**DOI:** 10.3390/polym14153210

**Published:** 2022-08-06

**Authors:** Victor S. Cecon, Greg W. Curtzwiler, Keith L. Vorst

**Affiliations:** 1 Polymer and Food Protection Consortium, Iowa State University, 536 Farm House Ln, Ames, IA 50011, USA; 2 Department of Food Science and Human Nutrition, Iowa State University, 536 Farm House Ln, Ames, IA 50011, USA

**Keywords:** crystalline poly(ethylene terephthalate), post-industrial recycling, polymer processing, biofillers, coffee chaff, rice hull

## Abstract

As the demand for plastics only increases, new methods are required to economically and sustainably increase plastic usage without landfill and environmental accumulation. In addition, the use of biofillers is encouraged as a way to reduce the cost of the final resin by incorporating agricultural and industrial waste by-products, such as rice hulls and coffee chaff to further reduce waste being sent to landfills. Crystalline poly(ethylene terephthalate) (CPET) is a resin commonly used for microwave and ovenable food packaging containers that have not been fully explored for recycling. In this article, we investigate how the incorporation of biofillers at 5% wt. and 10% wt. impacts critical polymer properties. The thermal and mechanical properties were not significantly altered with the presence of rice hulls or coffee chaff in the polymer matrix at 5% wt. loading, but some reduction in melt temperature, thermal stability, and maximum stress and strain was more noticed at 10% wt. The complex viscosity was also reduced with the introduction of biofillers. The levels of heavy metals of concern, such as Cd, Cr, and Pb, were below the regulatory limits applicable in the United States and Europe. Additional studies are suggested to improve the performance of CPET/biofiller blends by pre-treating the biofiller and using compatibilizers.

## 1. Introduction

Plastics are global and found everywhere, from cars to kitchen utensils and especially in packaging materials. To meet the demands for these applications, worldwide plastic production has been increasing over the years, reaching 367 million tonnes in 2020 [1]. Packaging applications are the segment where plastics are mainly used, with more than 40% of plastic volume in Europe [1]. Food packaging has received a lot of attention from environmental and regulatory agencies due to the increased use and waste accumulation of single-use plastics (SUP) from the COVID-19 pandemic [2], which include takeout and ready-to-cook (RTC) food products that are commonly microwave heated. In addition to the basic concept of containing and protecting the food, microwavable packages are required to meet more sophisticated performance requirements such as high-temperature cooking for convenience products. These performance requirements create challenges and potential interaction with the food resulting in the migration of chemicals from the packaging and/or discoloration [3].

To meet the performance requirements of high heat convenience packaging the most common configuration consists of thermoformed trays made out of crystalline poly(ethylene terephthalate) (CPET). These trays are considered “dual ovenable” due to their ability to be used in both conventional and microwave ovens [4,5]. CPET is a semi-crystalline polymer of elevated crystallinity, a result of its linear macromolecular structure that is produced from PET with the addition of nucleants [6]. These crystallization agents can include ultrafine inorganic powders with concentrations up to 0.3% wt. that work as a nucleating agent and polyolefins, such as polypropylene (PP), high-density polyethylene (HDPE), or linear low-density polyethylene (PE-LLD) in concentrations close to 3% wt. acting as “crack stoppers” [5]. Despite the downside of losing transparency, the resulting trays made with thermoformed CPET have increased mechanical stability and light protection, and can be used between −40 and 220 °C, allowing its use from freezer storage to heating in a microwave oven [7].

Recently, natural fillers, or biofillers, have been gaining attention as a modifier in polymer composites, considering the low cost and great availability, as well as potentially increasing the performance of the polymers mixed with them, including rice hull (or rice husks) and coffee chaff (or coffee silverskin) [8,9,10]. Rice hull is a significant agricultural waste by-product from the rice milling that consists of up to 20% wt. of the rice grain, with annual production consisting of up to 151 million tonnes, based on the 756 million tonnes of rice harvested globally in 2020 [11,12]. Coffee chaff is a thin skin that covers green coffee beans and are produced as a by-product from the roasting process, representing approximately 4% wt. of the coffee bean [10,13,14], potentially resulting in annual production of almost 400,000 tonnes, considering that 9.9 million tonnes were harvested globally in 2020 [15]. The utilization of these biofillers have been reported in the literature for several polymer composites, with recent uses including both fossil fuel-based polymers as polypropylene (PP) [13,16,17], polyamides (PA) [12], polyethylene (PE) [18,19,20], and poly(ethylene terephthalate) (PET) [21], as well as biopolymers such as poly (lactic acid) (PLA) [22,23], poly (butylene succinate) (PBS) [24,25,26,27,28], and poly(butylene adipate-co-terephthalate/poly(3-hydroxybutyrate-co-3-hydroxyvalerate) (PBAT/PHBV) [29,30].

Considering the widespread utilization of CPET and the need to increase its sustainability appeal, biofillers are proposed to be mixed with recycled CPET trays. Not only reducing packaging costs, biofillers such as coffee chaff and rice hulls are potentially cheaper than PET resin. Therefore, substituting CPET polymers with very low cost fillers such as coffee chaff and rice hulls presents a sustainable message to brand owners and consumers while replacing and reducing the use of fossil fuel-based feedstocks and potentially improving the chemical recycling of the blends [31]. Coupled with biofillers, the use of regrind or post-industrial recycled (PIR) CPET also provides a low-waste approach by limiting the amount of CPET scraps bound to waste-to-energy (WTE) facilities or, even worse, landfills, considering that plastic production scraps could reach up to 40% of total production [32]. The recycling of post-consumer CPET must be considered as multiple large fast-moving consumer goods (FMCG) companies, such as Nestle, PepsiCo, and Unilever, have set ambitious targets of increasing post-consumer recycled content in their plastic packaging in the next few years [33]. In addition, the United States Environmental Protection Agency (U.S. EPA) has recently set a goal of increasing the national recycling rate to 50% by 2030 [34], which will require significant effort from all stakeholders in the section to achieve it, considering that only 8.7% of plastics were recycled from municipal solid waste in 2018 [35]. However, multiple challenges exist when considering the difficulty separating CPET from PET with the current recycling infrastructure and eventual contamination of PET feedstock streams.

In this work, coffee chaff and rice hulls were compounded with regrind CPET trays in two different ratios of 5 and 10% wt. The study’s goal is to understand eventual changes that can be caused by the addition of the biofillers in the CPET properties, as few studies have investigated the reuse of CPET and there is greater attention towards the development of biocomposites [36,37]. The resulting blends were characterized and compared with the CPET without biofiller in terms of morphological, thermal, mechanical, and physical polymer properties. The presence of regulated heavy metals for food packaging was also investigated, as nineteen states in the United States limit the total incidental concentration of Cd, Cr^6+^, Hg, and Pb to 100 ppm in any finished package or packaging component [38]. The results of this work suggest the addition of coffee chaff or rice hulls did not drastically affect the performance of CPET blends, but some slight reductions in thermal and mechanical properties were observed at the highest concentration (10% wt.) of biofiller tested. The use of coffee chaff and rice hulls can be a solution to reduce costs for packaging manufacturers while reducing the use of fossil fuel-based resins and diverting agricultural and industrial waste byproducts.

## 2. Materials and Methods

### 2.1. Preparation of Biofiller-CPET Blends

**Figure 1:**
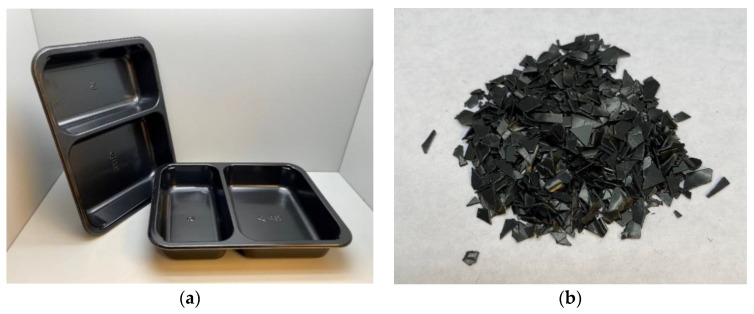
CPET (**a**) trays and (**b**) flakes used in CPET/biofiller blends.

**Figure 2:**
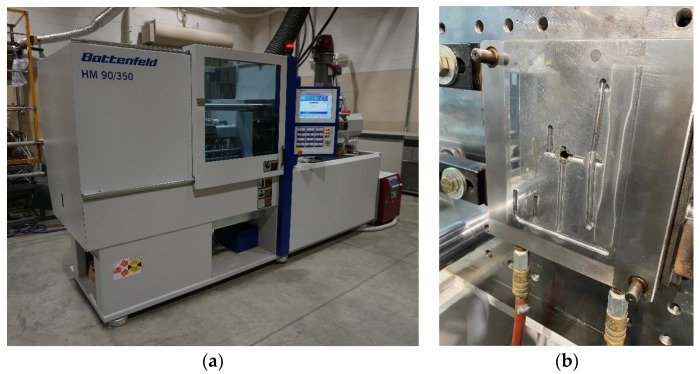
(**a**) Injection molder and (**b**) mold setup used in the production of Type I Dog Bone.

Crystalline poly(ethylene terephthalate) (CPET) was obtained from a packaging company in the form of black trays for food contact applications and then granulated into flakes using a commercial JS AO-10 granulator (Jumbo Steel Machinery, Taiwan), as shown in Figure 1. Coffee chaff (CC) and rice hulls (RH) were obtained from domestic sources from a coffee roaster and an agricultural company, respectively, as waste by-products. Blends containing 5% and 10% wt. of the biofillers were prepared using a Micro 18GL 18 mm twin-screen co-rotating extruder (Leistritz, Somerville, NJ, USA) and pelletized with a lab-scale pelletizer BT 25 (Bay Plastics Machinery, Bay City, MI, USA)., with the composition of each blend listed in Table 1. ASTM D638-14 injection-molded Type I dog bones were prepared using a production-grade HM90/350 90-ton horizontal injection molder (Wittmann Battenfeld, Torrington, CT, USA), as shown in Figure 2. Extrusion and injection molding feeds were flushed with N_2_ to minimize CPET degradation due to atmospheric moisture. CPET feedstock was oven-dried at 120 °C for 24 h prior to processing to remove eventual moisture. Processing parameters are listed in Table 2.

### 2.2. Microscope Imaging and Particle Size Analysis

Biofiller and injection molded (cryofractured) samples were analyzed using a 3D Surface Profiler VK-X1000 microscope (Keyence Corporation of America, Itasca, IL, USA). Biofiller images were captured at 5× magnification while injection molded samples were analyzed at 20× magnification. The particle size distribution of coffee chaff and rice hull were determined using a Mastersizer 2000 (Malvern Pananalytical, Worcestershire, UK), with the size range between 0.375 to 1000 µm.

### 2.3. Thermogravimetric Analysis

Thermal degradation properties such as the temperature of 5% mass loss and the ash residue of each blend of CEPT/biofiller were assessed through thermogravimetric analysis (TGA) using a Q5000IR thermogravimetric analyzer (TA Instruments, New Castle, DE, USA). For each CPET/biofiller blend, 5–10 mg specimens were weighted and loaded to a platinum pan, being heated at 10 °C/min up to 600 °C, under an N_2_ atmosphere.

### 2.4. Differential Scanning Calorimetry

Thermal transitions were analyzed through a heat/cool/heat cycle between −30 °C and 310 °C at a rate of 10 °C/min under an N_2_ atmosphere using a Q2000 differential scanning calorimeter (TA Instruments, New Castle, DE, USA). Each CPET/biofiller had specimens of 3–7 mg weighted and added into a hermetically sealed aluminum DSC pan that was crimped before the analysis.

### 2.5. Electromechanical Testing

Mechanical properties were assessed using an Autograph AGS-J (Shimadzu Corp., Kyoto, Japan) universal electromechanical tester with a 5 kN load cell and a manual non-shift wedge grip set MWG-5kNA (Shimadzu Corp., Kyoto, Japan) in the tensile mode. The specimens were evaluated for each CPET/biofiller blend following ASTM D638-14 with a 50 mm/min crosshead speed.

### 2.6. Fourier Transform Infrared Spectroscopy

Spectrometric analysis through Attenuated Total Reflectance Fourier Transform Infrared Spectroscopy (ATR-FTIR) was carried out using a Nicolet 6700 infrared spectrometer (Thermo Fisher, Waltham, MA, USA) at ambient temperature (22 °C) equipped with a DTGS detector. Each measurement had 32 scans and used a resolution of 2 cm^−1^, with the ATR accessory cleaned with an isopropanol wipe after each run to prevent cross-contamination. Spectra were analyzed with OMINIC^TM^ 8.3 software (Thermo Fisher, Waltham, MA, USA) to assess eventual interactions between the CPET and the biofillers.

### 2.7. Inductively Coupled Plasma—Optical Emission Spectroscopy

The presence of metals was evaluated using Inductively Coupled Plasma—Optical Emission Spectroscopy (ICP-OES). Specimens of 0.1500 ± 0.0005 g were weighted for each CPET/biofiller blend and digested via microwave-assisted digestion using an UltraWave digestion system (Milestone, Inc., Shelton, CT, USA) in 5 mL HNO_3_ 67% *v/v* Trace Metal Grade (Fisher Scientific, Fair Lawn, NJ, USA) and 1 mL HCl 34% *v/v* Trace Metal Grade (Fisher Scientific, Fair Lawn, NJ, USA). An initial pressure of 40 bar (N_2_) was applied, as well as a microwave power of 1350 W for 45 min (with a ramp of 5 min), from room temperature (22 °C) to 210 °C with a cooling time of 20 min. Initially, a pressure of 40 bar with N2 was applied, followed by the use of a microwave power of 1500 W for 40 min, with a temperature increase from room temperature (22 °C) to 260 °C and a system pressure increase to 150 bar. A cooling period of 20 min was then applied. The method used is a slight modification of the modified Westerhoff digestion method described by Goodlaxson [39,40].

After the microwave digestion, the samples were diluted to 50 mL with ultra-pure, deionized water and analyzed using an iCap-7400 Duo ICP-OES (Thermo Scientific, Waltham, MA, USA). The analysis was carried out using the wavelength with the lowest limit of detection (LOD) for each metal. All measurements were performed in radial mode. Multi-element standard solutions containing Al (aluminum), Cd (cadmium), Cr (chromium), Fe (iron), Pb (lead), Sb (antimony), and Ti (titanium) were prepared for the concentrations of 0.1 µg/mL, 1 µg/mL, 5 µg/mL, 25 µg/mL, 50 µg/mL, and 100 µg/mL of each metal of interest, along with a 5 µg/mL yttrium internal standard. They were prepared using single standard solutions of 1000 µg/mL (Inorganic Ventures, Christiansburg, VA, USA) for each metal. Concurrent blanks (with no polymer added) were also run for each digestion batch for metals eventually present in the acids or leached from digestion vessels.

### 2.8. Parallel-Plate Oscillatory Melt Rheometry

Rheological analysis of the CPET/biofiller blends was carried out in oscillatory mode (frequency sweep) using a DHR-2 hybrid rheometer (TA Instruments, New Castle, UK). The rheometer was equipped with an environmental test chamber and a 25 mm parallel-plate geometry with a 1 mm gap between plates was used. All tested samples were dried in a convection oven at 140 °C overnight prior to analysis. The measurements were conducted within the linear viscoelastic region (previously tested) under 1% strain, at 270 °C, and air atmosphere, with the angular frequency ranging from 0.05 to 500 rad/s.

### 2.9. Statistical Analysis

Statistical analysis was conducted with a one-way ANOVA considering a 95% confidence level (α = 0.05) and grouped using Tukey’s honestly significant difference (HSD) test. The JMP^®^ 15 Pro (SAS Institute Inc., Cary, NC, USA) was utilized as the statistical analysis software. All analyses used five repeated measures unless otherwise noted.

## 3. Results and Discussion

### 3.1. Biofiller Characterization and Sample Imaging

**Figure 3:**
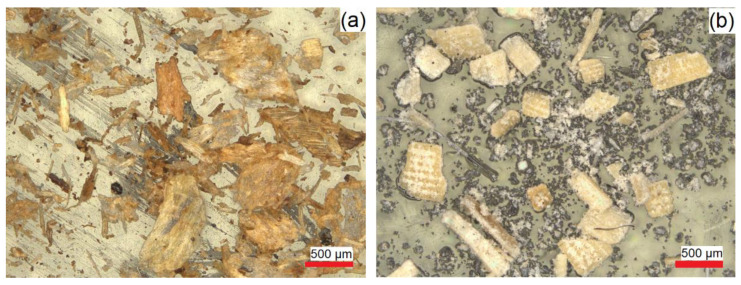
Optical images of the biofillers: (**a**) coffee chaff; (**b**) rice hull.

**Figure 4:**
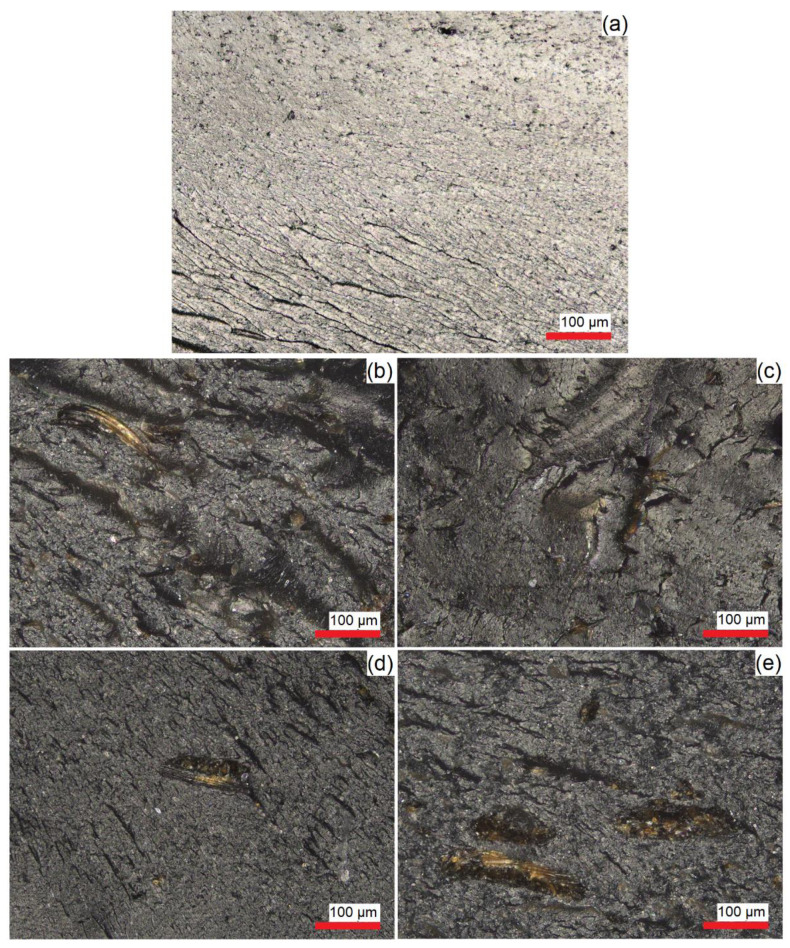
Optical images of cryofractured injection molded samples: (**a**) no biofiller; (**b**) 5% wt. coffee chaff; (**c**) 10% wt. coffee chaff; (**d**) 5% wt. rice hull; (**e**) 10% wt. rice hull.

The optical images of coffee chaff and rice hull are displayed in Figure 3, where a heterogenous particle size distribution and anisotropic particles can be observed. Coffee chaff particles are darker than rice hull particles and are wafer-like, while rice ones have a flake-like morphology. From Figure 4, it is shown that the biofillers are well dispersed in the matrix, with no agglomeration observed. The presence of the biofillers was noticed by the brown spots localized in the polymer matrix with different sizes, as the biofillers presented a large particle size distribution.

**Figure 5:**
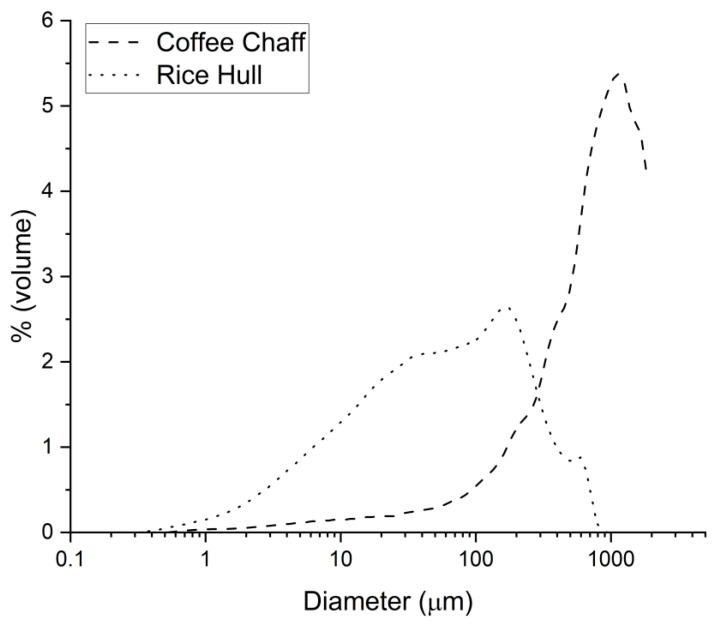
Particle size distribution for coffee chaff (dashed line) and rice hull (dotted line).

The particle size analysis showed that coffee chaff, with a median size of 780.8 µm, had a larger particle size than rice hull, with a median size of 58.9 µm. Due to the equipment limitation on the maximum particle size range, sizes above 2000 µm were not recorded for coffee chaff, and the actual numbers would potentially indicate a larger median particle size. This result, illustrated by the particle size distribution in Figure 5, correlates with Figure 3, where it can be seen that coffee chaff has larger particles than rice hull and has a wider distribution.

### 3.2. Impact of Biofillers on the Thermal Properties

**Figure 6:**
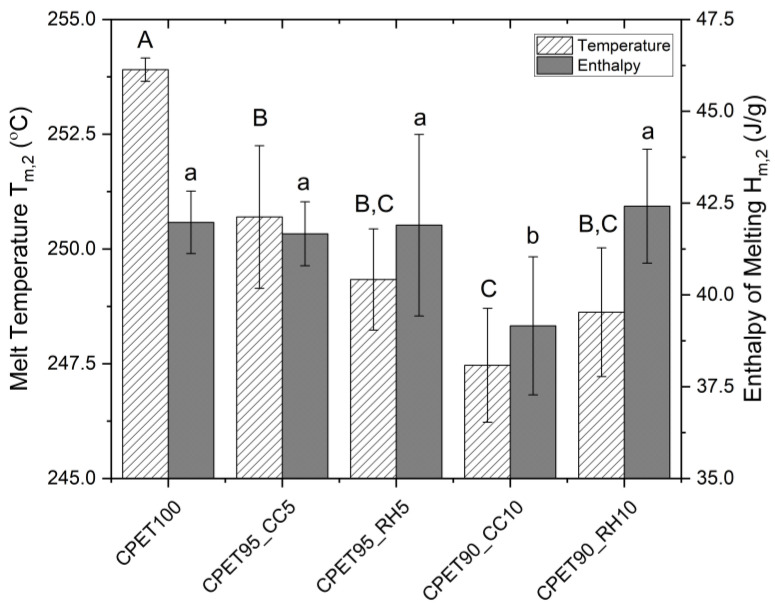
Melt temperature and enthalpy of melting from the second heat cycle. Data points associated with the same letter are statistically equivalent (α = 0.05); uppercase for melt temperature, lowercase for enthalpy.

Differential scanning calorimetry was performed to understand how the addition of biofiller influences the melt temperature and enthalpy of melting compared to CPET as it relates to thermal stability in rapid heating applications (microwave/oven). It was observed that adding both coffee chaff or rice hulls to CPET caused a statistically significant decrease (*p* < 0.05) in the melt temperature, as shown in Figure 6, although not enough to considerably change the polymer application. The increase from 5 to 10% wt. on the coffee chaff loading produced a significant decrease in temperature, whereas the two concentrations of rice hull tested did not yield a significant difference (*p* < 0.05). The enthalpy was consistent for all samples with the exception of the blend containing 10% wt. of coffee chaff, which displayed a statistically lower value. This result suggests that the biofillers do not act as a nucleant for CPET and additional studies are required to investigate the possible substitution of the inorganic nucleation agents by biofillers, considering that their particle size (not measured in this study) might have an important role. When taking into account only these two parameters, the blends containing rice hull displayed a lower deviation from the 100% CPET samples than coffee chaff.

**Figure 7:**
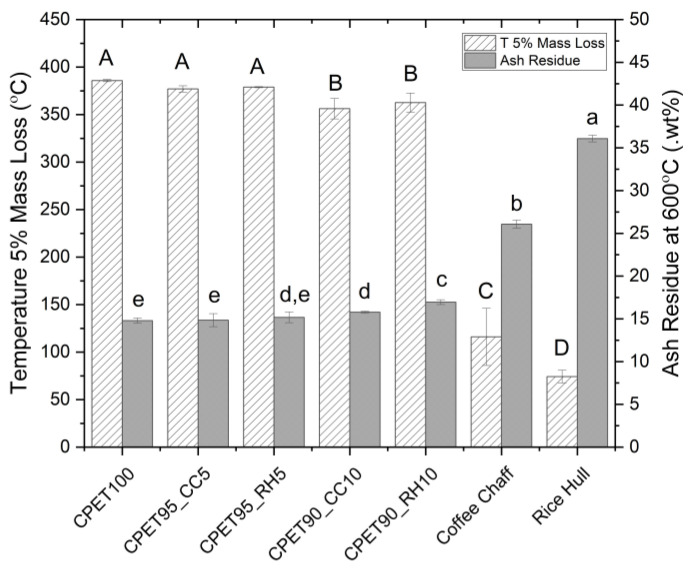
The temperature at 5% mass loss and ash residue at 600 °C. Data points associated with the same letter are statistically equivalent (α = 0.05); uppercase for temperature, lowercase for ash residue.

The results from the thermogravimetric analysis in Figure 7 showed that at 5% wt., both biofillers did not significantly reduce the temperature at 5% mass loss, while the decrease was more noticeable when coffee chaff or rice hulls were mixed at 10% wt. The ash residue at 600 °C generally increased with more biofiller added to the blend, but there was no significant difference when coffee chaff was added at 5% wt. The analysis of the pure biofillers showed higher temperature and lower ash residue for coffee chaff when compared to rice hull, but the effect was not noticed in the CPET/biofiller blends analyzed. A review by Suhot, et al. [41] analyzed several publications discussing the use of rice husk in different polymers and found both positive and negative impacts on the composites’ thermal properties made of polypropylene (PP) or low-density polyethylene (PE-LD), for example. A possible alternative to improve thermal stability is using halogen-free flame retardants to increase the fiber-matrix interaction, as observed in the work of Phan, et al. [42]. Aluminum diethylphosphinate and aluminum hydroxide were used as flame retardants in polyurethane composites and increased the temperature at 5% mass loss by 40 and 32 °C, respectively.

### 3.3. Impact of Biofillers on the Mechanical Properties

**Figure 8:**
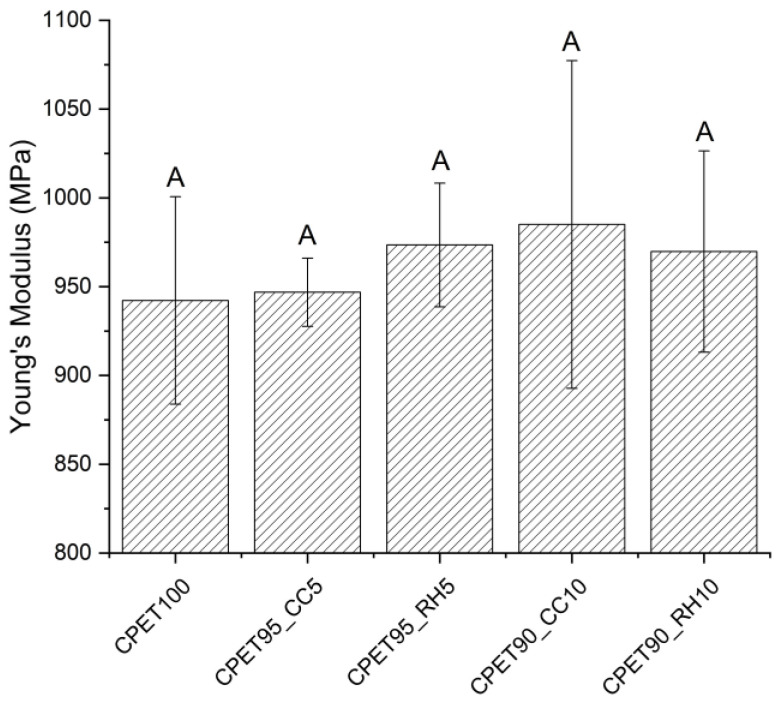
Young’s Modulus for the CPET/biofiller blends. Data points associated with the same letter are statistically equivalent (α = 0.05).

**Figure 9:**
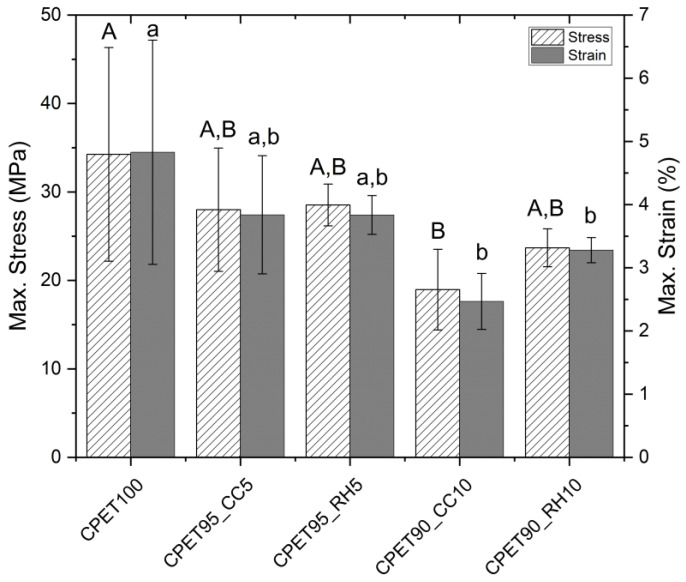
Maximum stress and strain for the CPET/biofiller blends. Data points associated with the same letter are statistically equivalent (α = 0.05); uppercase for stress, lowercase for strain.

A tensile test was performed to evaluate the mechanical properties of injection-molded specimens made of CPET/biofiller blends. From Young’s modulus results shown in Figure 8, it is possible to observe that there was no significant difference between all blends compared to the scenario without any biofiller, which can be correlated to the similar morphology observed in the cryofractured samples shown in Figure 4, despite the difference in biofiller particle size. However, from the maximum stress and strain results displayed in Figure 9, the blend containing 10% wt. of coffee chaff is the only blend that is significantly different from the 100% CPET samples for both stress and strain, with lower values, possibly due to larger particle size than rice hull. The blend with 10% wt. rice hulls also presented a strain value significantly different from the control scenario, being statistically equivalent to the 10% wt. coffee chaff blend. The samples containing 5% wt. of biofiller were statistically equivalent to 100% CPET, and the blends with 10% wt. biofiller.

Testing rice husks concentrations between 10% wt. and 30% wt. with polypropylene (PP), Hidalgo-Salazar and Salinas [17] observed a significant increase in the tensile modulus as more biofiller was present in the blend. A study using recycled polyethylene (rPE) and poly(ethylene terephthalate) (rPET), blended at 75% rPE/25% rPET and compounded with rice husk in concentrations between 40% wt. and 80% wt. found that Young’s modulus increased up to 70% wt. of rice husk added to the blend, with a sharp decrease at 80% wt [21]. Another blend containing 80% wt. PET and 20% wt. linear low-density polyethylene (PE-LLD) recovered from water bottles and used bale wrap, respectively, was mixed with a biocarbon, made of pyrolyzed spent coffee ground in concentrations between 5% wt. and 20% wt. Gupta, et al. [43] found increased tensile modulus with increased biofiller content; however, the ultimate tensile strength (UTS) and impact resistance presented an opposite response with decreased performance with more biocarbon. A direct comparison with these prior two studies cannot be made considering the different resins utilized and the use of compatibilizers. The pre-treatment of the rice hull and coffee chaff, as observed by Suhot, Hassan, Aziz, and Md Daud [41] for composites containing rice husks, and the use of a coupling agent would be beneficial for the mechanical performance of the CPET/biofiller blends. This is particularly useful when considering that CPET commonly has the presence of inorganic nucleation agents and “crack stoppers”, as previously discussed.

### 3.4. Spectrometric Analysis of CPET/Biofiller Blends

**Figure 10:**
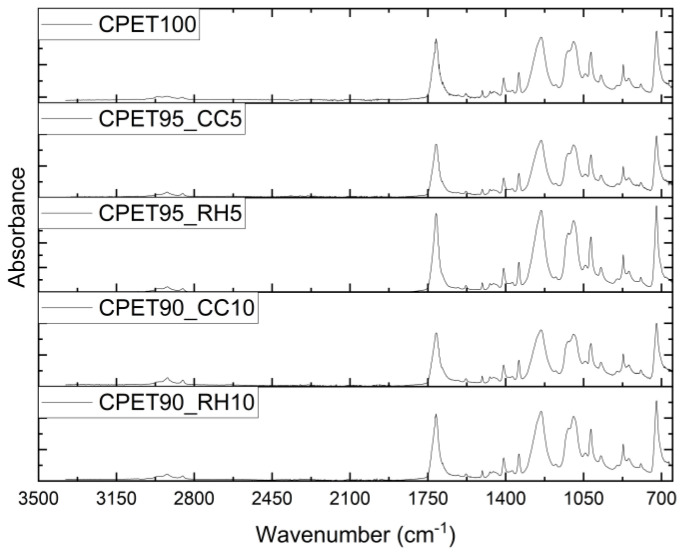
ATR-FTIR spectra for CPET/biofiller blends.

The ATR-FTIR analysis did not find any new vibrational peak present with the addition of coffee chaff or rice hull, as shown in the spectra displayed in Figure 10. The main absorbance peaks observed are related to CPET, including at 1715 cm^−1^ for ν-C = O (stretching, ester, ring), 1240 cm^−1^ for ν-C(=O)-O (stretching), ν-C-C (ring, ester, stretching), and δ-C-H (ring, in-plane bending), 1095 cm^−1^ for ν-C-O (stretching), 1017 cm^−1^ for ν-C-O (stretching), 871 cm^−1^ for δ-C-H (ring, out-of-plane bending), δ-C-C (ring, ester, out-of-plane bending), and δ-C = O (out-of-plane bending, ring torsion), and 721 cm^−1^ for δ-C = O (out-of-plane bending), and δ-C-H (ring, out-of-plane bending) [44,45]. Additionally, no frequency shift or alteration in peak shape was noticed when the biofillers were introduced into the polymer blend, suggesting that there was no change in the intermolecular interactions. For example, it was reported in the literature that rice husks, which contain 29–35% of cellulose and 19–26% of lignin, could alter peaks at 3421 cm^−1^ (O-H bonds of lignin) and 1280 cm^−1^ (C-H bonds of crystalline parts of cellulose) [46]. Still, such an effect was not noticed in the CPET blends, possibly due to the low biofiller loading.

### 3.5. Rheological Analysis

**Figure 11:**
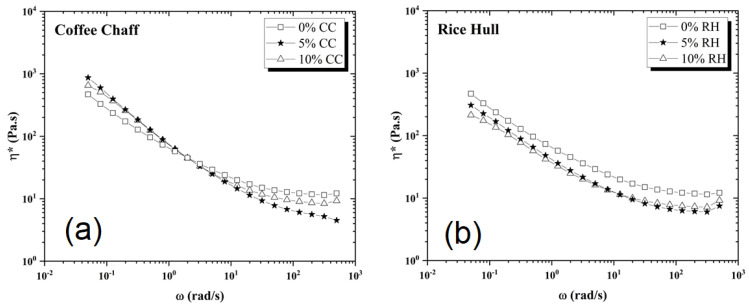
Complex viscosity plots of blends containing (**a**) coffee chaff and (**b**) rice hull at 270 °C.

The impact of biofillers on the sample viscosity was assessed using oscillatory melt rheology tests, as it could yield differences in polymer processing. It was noticed from the complex viscosity results, shown in Figure 11, that the addition of coffee chaff or rice hull can reduce the viscosity at higher frequencies for both biofillers. For coffee chaff, the viscosity increased at lower frequencies (<1 rad/s) with the addition of the biofiller, with 5% wt. displaying both the highest increase and the decrease at lower and higher frequencies, respectively. For rice hull, at 5% wt. and 10% wt. there was a reduction in viscosity, with the latter presenting a more noticeable decrease at lower frequencies while the 5% wt. blend had the smallest complex viscosity at higher frequencies. The results suggest that the incorporation of 5–10% wt. of coffee chaff or rice hull can reduce the viscosity of the final blend under certain processing conditions. In the blend of pyrolyzed coffee chaff with PET and PE-LLD, Gupta, Mohanty, and Misra [43] found that the complex viscosity increased when the biofiller was at 5% wt., followed by a decrease at higher concentrations. It was suggested that the biofiller could have prevented the entanglement and sliding of chains, which reduced the complex viscosity and could have happened at 5% wt. and 10% wt. in this study.

We hypothesize that solid-state polymerization (SSP) and the use of a chain extender, as done for PET, might be beneficial to improve the melt behavior of the reprocessed CPET, as the thermal reprocessing of the material could lead to a molecular weight reduction and consequently, cause a reduction in mechanical and rheological properties [47,48]. Also, the addition of a compatibilizer might enhance the mixture between the biofillers and the CPET, having the potential to increase the availability of carboxylic or hydroxyl end groups from the biofillers to promote the esterification and transesterification reactions of SSP, and thus, increase the molecular weight of the reprocessed CPET.

### 3.6. Metal Analysis of CPET/Biofiller Blends

The metal analysis using ICP-OES was performed to assess the potential use of the CPET/biofiller blends for packaging applications, considering that multiple states in the United States have limits on the heavy metal content and main application of CPET for “dual-ovenable” use. The analysis did not find the presence of Cd and Pb (Hg was not tested but historically is not detected), and the amounts of Al, Cr, Fe, and Sb were summarized in Table 3. The samples would comply with the Toxics in Packaging legislation [38] in the United States and with Article 11 of the European Parliament and Council Direction 94/62/EC [49], limiting to 100 ppm the combined concentration of Cd, Cr^6+^, Hg, and Pb, as only Cr was detected for the samples containing rice hulls, but at 2.58 and 5.32 ppm for 5% wt. and 10% wt. of the biofiller, respectively. The other metals found are commonly found in plastics due to its use as a catalyst during the polymerization process or as thermal and chemical stabilizers [50].

## 4. Conclusions

This work presented the impact on the polymer properties of adding coffee chaff and rice hulls to regrind CPET, being an alternative for plastic manufacturers to comply with consumer and government requests to reduce plastic use in packaging products while keeping costs down as fossil fuel-based plastic resins continue to rise based on oil prices and the low availability of recycled resins [51]. The analysis of the biofillers showed that coffee chaff presented higher particle size when compared to rice hull, but the images from the cryofractured blend samples did not show significant differences in morphology. Thermal analysis showed that blends containing 10% wt. of biofiller had a greater impact, with the reduction of melt temperature, enthalpy, and temperature at 5% mass loss being more significant when compared to the blend with only 5% wt. of coffee chaff or rice hull. When comparing both biofillers, the latter performed slightly better, with the enthalpy for the 10% wt. blend being statistically equivalent to the 5% wt. blend, which was not the case for coffee chaff.

There was no statistical difference between all samples for Young’s modulus, although the maximum stress and strain results showed decreased performance for the blends containing 10% wt. of biofiller. The presence of coffee chaff or rice hull both at 5 and 10% wt. reduced the complex viscosity for the CPET blends, which could be potentially improved by the use of a compatibilizer. No noticeable difference was noticed in the spectra of the samples with or without biofiller, possibly due to the low loading of coffee chaff and rice hull. The metal analysis did not find levels of regulated metals (Cd, Cr, and Pb) above the limits established by legislation in the United States and Europe for packaging products.

Most of the literature reports the mixture of biofillers with polyolefins, with limited works involving the use of PET. Considering multiple applications of this resin for transparent products, biofillers could have an impact on these characteristics, which is not the case for CPET, a subtype of PET with also limited published research. The use of biofillers in recycled plastics helps to reduce landfill accumulation from both a traditional petroleum-based product (CPET) as well as industrial and agricultural by-product waste (coffee chaff and rice hull), contributing to a more circular economy and potential reduction of greenhouse gases from the production of virgin feedstocks as less virgin resin is used when the biofiller is incorporated. Despite the slight decrease in performance observed, this work expands the knowledge on CPET reuse, warranting additional studies to investigate the potential use of compatibilizers or the pre-treatment of the biofillers with the goal of improving the performance of blended CPET/biofiller package products. Future research should also look to understand the impacts of biofiller on product application. Using coffee chaff as an example, one could impart a characteristic odor to the final product made with the CPET/biofiller blend, and the utilization of techniques to improve molecular weight and viscosity of reprocessed CPET, as current literature is focused on the implications for PET.

## Figures and Tables

**Table 1:** CPET/biofiller blends formulation in terms of weight percentage.

Sample Name	CPET	Biofiller Type	Biofiller Content
CPET100	100	-	-
CPET95_CC5	95	Coffee Chaff	5%
CPET90_CC10	90		10%
CPET95_RH5	95	Rice Hull	5%
CPET90_RH10	90		10%

**Table 2:** Processing parameters of CPET/biofiller blends.

Extrusion	CPET100	CPET95 CC5	CPET90 CC10	CPET95 RH5	CPET90 RH10
Zone 1 Temperature (°C)	200	200	200	200	200
Zone 2 Temperature (°C)	265	250	250	265	250
Zone 3 Temperature (°C)	270	255	255	265	255
Zone 4 Temperature (°C)	270	255	255	270	255
Zone 5 Temperature (°C)	275	260	255	275	260
Zone 6 Temperature (°C)	285	260	255	280	260
Zone 7 Temperature (°C)	295	260	250	285	265
Die Temperature (°C)	300	255	245	285	265
**Injection Molding**					
Feed Zone Temperature (°C)	49				
Zone 1 Temperature (°C)	265				
Zone 2 Temperature (°C)	260				
Zone 3 Temperature (°C)	250				
Nozzle Temperature (°C)	285				

**Table 3:** Inorganic element composition of selected compounds (Al, Cr, Fe, Sb) in CPET and blends containing coffee chaff or rice hull as biofillers.

Sample Name	Al (ppm)	Cr (ppm)	Fe (ppm)	Sb (ppm)
CPET100	50.92	*b	43.41	168.61
CPET95_CC5	87.07	*b	53.61	159.13
CPET90_CC10	15.87	*b	51.12	96.75
CPET95_RH5	*a	2.58	33.55	162.41
CPET90_RH10	*b	5.32	86.26	160.53
LOD (ppm)	0.0101	0.0009	0.0015	0.0156
LOQ (ppm)	0.0337	0.0030	0.0050	0.0520

*a: value between LOD and LOQ; *b: value below LOD.

## Data Availability

The data in this study may be available upon request from the corresponding author.
